# Assessment of tumor microenvironment expression and clinical significance of immune inhibitory molecule CTLA-4, ligand B7-1, and tumor-infiltrating regulatory cells in Hodgkin lymphoma

**DOI:** 10.25122/jml-2023-0019

**Published:** 2023-04

**Authors:** Radhey Shyam Verma, Gyanendra Singh, Anurag Singh, Pradyuman Singh

**Affiliations:** 1 Department of Pathology, Maharaja SuhelDev Autonomous State Medical College, MaharshiBalark Hospital, Bahraich, Uttar Pradesh, India; 2 All India Institute of Medical Sciences Rajkot, Gujarat, India; 3 Department of Pathology, King George's Medical University, Lucknow Uttar Pradesh, India; 4 Department of Pathology, Dr Ram ManoharLohia Institute of Medical Sciences, Lucknow Uttar Pradesh, India

**Keywords:** Hodgkin's lymphoma, Hodgkin Reed-Sternberg cell, CTLA4, CD80 (B7-1), tumor microenvironment

## Abstract

Classical Hodgkin lymphoma represents a paradigm of tumor cell–microenvironment interactions as the neoplastic Hodgkin Reed–Sternberg (HRS) cells typically constitute less than 1% of the total tumor volume. CTLA-4, a member of the CD28/B7 immunoglobulin superfamily, and CD28 and their ligands B7-1 and B7-2 are critically important for the initial activation of naive T cells. Strategies aimed at interfering with the crosstalk between tumoral Reed-Sternberg cells and their cellular partners have been taken into account in the development of new immunotherapies that target different cell components of the HL microenvironment. The study included 50 histopathological confirmed cases of Hodgkin lymphoma. IHC staining for CTLA-4 and B7-1 was performed on archival paraffin-embedded biopsy. SPSS version 17 was used for statistical analysis. CTLA-4 IHC expression in HRS cells was negative in all cases, while in immune cells, CTLA-4 expression was observed in 45 (90%) cases. CD80 expression was present in all cases, both in HRS and immune cells. There was a significant association between HRS cell percentage and IPS score (p-value=0.001). Mean survival duration was longer in <50% immune cells compared to >50% groups, with an overall mean survival of 67.633 months. Considering the CTLA4 expression in immune cells within the microenvironment and the availability of targeted drugs like Iplimumab, which act through CTLA4 blockade, it may be appropriate to use this as targeted therapy in HL cases, particularly in those with refractory disease who are unable to achieve cure prior to ASCT.

## INTRODUCTION

Classical Hodgkin lymphoma (cHL) represents a paradigm of tumor cell–microenvironment interactions, as the neoplastic Hodgkin Reed–Sternberg (HRS) cells typically constitute less than 1% of the total tumor volume. These cells are surrounded by a non-neoplastic (immune cell) infiltrate of T and B cells, eosinophils, neutrophils, plasma cells, histiocytes, fibroblasts, and extracellular matrix (ECM), making cHL a good model for studying the influence of the tumor microenvironment on cancer pathogenesis [1,2].

Recent developments in our knowledge of the biology and immunology of Hodgkin's lymphoma (HL) suggest that immune cells and cytokines infiltrated in the tumoral microenvironment may perform various roles closely related to clinical outcomes. HRS and inflammatory cells in the microenvironment secrete numerous cytokines and chemokines, enabling tumor cells to avoid immune cell attacks and grow [3-6]. The production and activation of T cells specific to tumor antigens are necessary for effective antitumor immunity. T-cell activation, proliferation, and the gain or loss of effector function are regulated by a variety of T-cell costimulatory receptors and T-cell negative regulators, also known as coinhibitory receptors. CD28 and CTLA-4 (cytotoxic T lymphocyte-associated antigen-4) are two of the earliest and best-studied co-stimulatory and co-inhibitory T-cell molecules [7].

CTLA-4, also known as CD152, is an immune regulatory molecule that belongs to the CD28/B7 immunoglobulin superfamily. CTLA-4, CD28, and their ligands B7-1 (CD80) and B7-2 (CD86) are critical for the initial activation of naive T cells as well as the regulation of the clonal composition of the responding repertoire after activated dendritic cells migrate to lymphoid organs [8, 9]. CTLA-4, involved in the activation of cytotoxic T cells, is expressed on activated T cells but not on resting T cells. CD28 transmits co-stimulatory signals that activate T-cell receptors by binding to B7-1 and B7-2 ligands on antigen-presenting cells, while CTLA-4 transmits a suppressive signal that inhibits T-cell growth and function [10].

Immune checkpoints that support physiological self-tolerance have been linked in recent years to the suppression of anti-tumor immunity. Novel immunotherapies that target various cell components of the HL microenvironment have emerged using approaches designed to disrupt the communication between tumoral Reed-Sternberg cells and their cellular partners. The different subtypes of HL have typical microenvironments, as the RS cells modify their surroundings to promote survival.

One potential strategy to improve anti-tumor immunity is the therapeutic blockade of immune inhibitory checkpoints. Programmed cell death protein 1 (PD-1) on T lymphocytes and its main ligand (PD-L1) on tumor cells are the targets of antibody-based interventions aimed at reactivating latent anti-tumor immunity. Preclinical research has shown that CTLA-4 controls the activation threshold and limits the proliferation of T lymphocytes specific to tumors. Antibodies that block CTLA-4 or PD-1/PD-L1 preferentially activate T cells targeting cancer cells [2]. Reversing T cell exhaustion and restoring immune response by blocking inhibitory molecules may help treat HL through immunotherapy.

Currently, no published data from India supports these findings in our patient population. Therefore, this study aimed to analyze immune inhibitory molecule CTLA-4 and B7-1 expression in the tumor cells and the microenvironment of Hodgkin lymphoma.

## MATERIAL AND METHODS

This study assessed the tumor microenvironment expression and clinical significance of the immune inhibitory molecule CTLA-4 and tumor-infiltrating regulatory cells in Hodgkin lymphoma.

### Objectives

1. To investigate the expression of immune inhibitory molecule CTLA-4 and its ligand B7-1 in tumors using immunohistochemistry (IHC).
2. To study the regulatory immune cells in the tumor microenvironment in relation to CTLA-4 and B7-1 using immunohistochemistry.
3. To correlate the expression of CTLA-4 and B7-1 with the disease characteristics, such as HL subtype, Ann Arbor stage, and IPS score.

### Study design

This was a retrospective and prospective analytical study conducted at the Department of Pathology of Dr. Ram ManoharLohia Institute of Medical Sciences, Lucknow. The study included histopathologically confirmed cases of Hodgkin Lymphoma diagnosed at our institute from April 2011 to November 2015 and undergoing treatment or under follow-up after completion of treatment. A total of 50 cases were included in the study.

### Inclusion and exclusion criteria

Inclusion criteria for our study comprised cases of histologically confirmed Hodgkin lymphoma with the availability of sufficient formalin-fixed paraffin-embedded (FFPE) tumor tissue and clinical details at presentation and follow-up. Exclusion criteria were insufficient FFPE tumor tissue, unavailability of clinical details at presentation, and inability to obtain consent.

### Case assessment

#### Histopathology review

All previously diagnosed cases were reviewed for confirmation of diagnosis and subtyping of Hodgkin Lymphoma.

**A. Review of medical records**

The medical records of the histologically confirmed cases at the initial presentation were reviewed for Ann Arbor staging, IPS, and treatment details.

**B. Special immunohistochemistry work-up**

IHC staining for CTLA-4 (CD152) and CD80 (B7-1) was performed on archival paraffin-embedded biopsy tissue with adequate tissue remaining after a routine case work-up. Immunohistochemical staining was performed on DAKO Autostainer using the commercially available antibodies and their corresponding detection systems.

**C. Collection of clinical data at follow-up**

Subjects were followed up retrospectively from their medical records and prospectively during regular follow-up outpatient department (OPD) visits. Any progression of disease, recurrence or relapse was noted. Progression-free survival (PFS) was measured from the date of diagnosis.

### Detailed Methodology

**Collection of clinical data and staging**

The clinical details were collected from the hospital information system (HIS) of RML IMS Lucknow or directly from the patients and entered into a case record form after obtaining informed consent. The clinical staging was done using the Ann Arbor staging system and International Prognostic Score (IPS) system.

### Immunohistochemistry for CTLA4 (CD152) and B7-1(CD80)

#### Anti-CTLA4 antibody

Mouse monoclonal antibody; clone BNI3, IgG2a isotype, Novus Biologicals USA.

Dilution: 1/200, optimized after standardization.

Retrieval was done in an alkaline medium in EDTA.

Control: Reactive lymph node.

#### Anti CD80 antibody

Rabbit monoclonal antibody, clone 62N3G8, IgG2b kappa isotype, Novus Biologicals USA.

Dilution: 1/200, optimized after standardization.

Retrieval was done in an alkaline medium, in EDTA.

Control: reactive lymph node.

The secondary antibody was polymeric HRP (Horse Radish Peroxidase) for CTLA4 and CD80.

#### Immunohistochemical evaluation

The immunohistochemical evaluation was performed using a polymeric Horse radish immunoperoxidase method. The primary antibody, Lyophilized Mouse Monoclonal CTLA4 Antibody clone BNI3, 1:200 dilution, and rabbit monoclonal CD80 antibody clone 62N3G8, 1:200 dilution was applied. A reactive node was used as a positive control for immunostaining.

#### IHC interpretation

**Figure 1:**
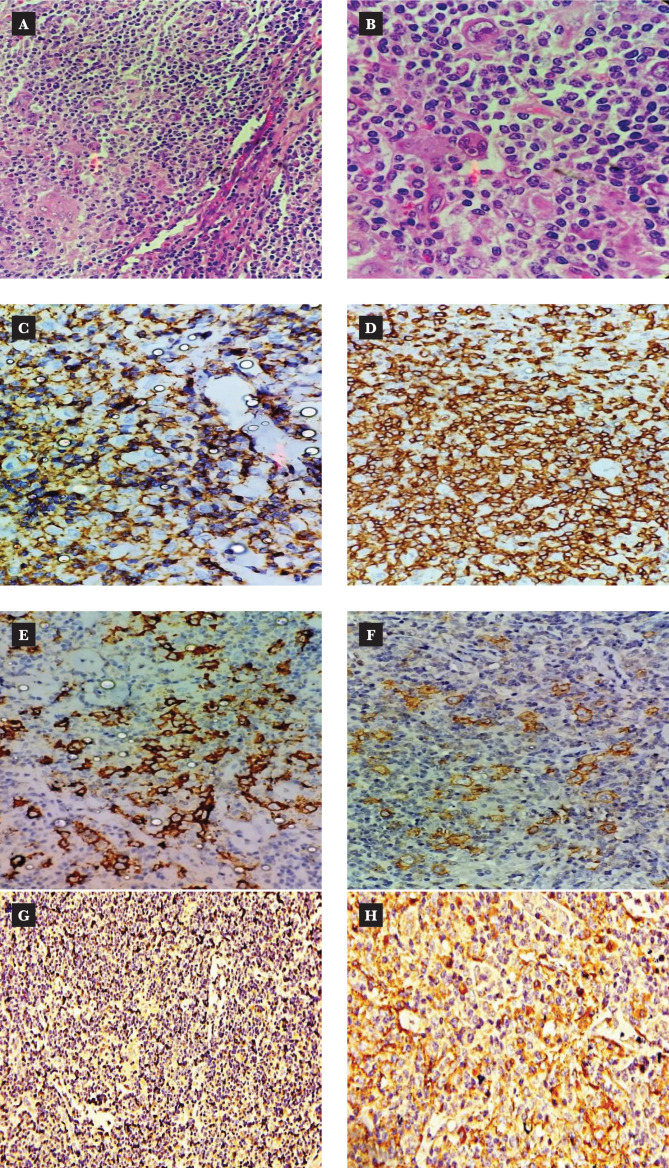
Classical Hodgkin lymphoma-lymphocyte rich subtype; A, B: H&E stain sections 200X and 400X respectively; C: LCA negative in HRS cells; D: CD3 positive in background lymphocytes; E: CD15, membranous and cytoplasmic positivity in HRS cells; F: CD30 membranous and Golgi zone positivity in HRS cells; G: CTLA-4 cytoplasmic positivity in immune cells; H: CD80 cytoplasmic positivity in HRS cells and immune cells.

Reactivity for both CTLA-4 and CD80 was determined by two pathologists by establishing a semi-quantitative score for each marker. The tumor component and non-tumor component (immune cells) were scored separately. For each case, the percentage expression of cells, pattern of expression, and intensity of staining of both CTLA-4 and CD80 IHC on tumor cells and non-tumor cells (immune cells) were determined. The pattern of staining was noted as cytoplasmic (C), membranous (M), cytoplasmic, and membranous both (C+M) or nuclear. The percentage expression for tumor cells and immune cells for both monoclonal antibodies was recorded as negative (0), less than 1% expression, 1-10%, 11-50%, and more than 50% expression. Staining intensity was graded as 0, 1+, 2+, and 3+ (Figure 1 A–H).

### Follow-up

All patients were seen for regular follow-up, and clinical data were collected from the hospital information system (HIS), medical records, and telephonic conversations with the patient and their close relatives. The aim was to collect retrospective data regarding the current health status of the patient, recurrence, and survival. The progression-free survival (PFS) was noted in all patients during follow-up. Overall survival (OS) was defined as the time between the diagnosis and death or the last follow-up. Patients still alive at the last follow-up were considered a censored event in the analysis.

### Statistical analysis

All data collected was recorded in the case record form and later entered into a Microsoft Excel sheet. Statistical analysis was performed using SPSS version 17. Continuous data were summarized as mean ± standard deviation (SD), while categorical variables were presented in numbers and percentages. Categorical groups were compared using the chi-square (χ^2^) test, and Kaplan-Meier survival analysis was performed to compare survival between the groups. A two-tailed (α=2) p-value less than 0.05 (p<0.05) was considered statistically significant.

## RESULTS

The study included 50 cases of Hodgkin Lymphoma diagnosed in our pathology department. Of these 50 cases, 42 (84%) were males and 8 (16%) females, with a mean age of 43.3 years (ranging from 5 to 71 years).

Table 1 shows the distribution of HL cases based on the histological subtypes. Most cases included in the study were mixed cellularity sub-type followed by lymphocyte-rich sub-types. Cervical lymphadenopathy was the most common initial presentation at the time of diagnosis, which was observed in 30 (60%) cases, followed by axillary lymphadenopathy in 8 (16%) cases. 2 (4%) cases showed mediastinal involvement.

**Table 1:** Distribution of HL cases included in the study as per WHO classification 2008

Types and subtypes of HL		No of cases (%)
**Nodular lymphocyte-predominant Hodgkin lymphoma**		1 (2%)
**Classical Hodgkin lymphoma (cHL)**	Mixed cellularity subtype (MC)	26 (52%)
	Lymphocyte-rich subtype (LR)	14 (28%)
	Nodular sclerosis subtype (NS)	09 (18%)
	Lymphocyte-depleted subtype (LD)	00 (0%)
**Total**		50

The Ann Arbor staging system was used to classify HL cases at the time of presentation. Most patients had presented in Ann Arbor stage II (16 cases; 32%), followed by stage III (11 cases; 22%). Stage IV comprised 8 cases (16%), stage I had 6 cases (12%), and 9 cases (18%) were unspecified.

The clinical staging of patients at the time of diagnosis was done using the Ann Arbor staging system, the International Prognostic Index/Score (IPI/IPS) system, or both. In our study, both methods were used for staging, and the clinical stage of the patient at presentation was recorded at the time of diagnosis. The staging records were available in 41 cases out of 50 cases. Most cases had presented in Ann Arbor stage II, 16 cases (32%), and Ann Arbor stage III, 11 cases (22%). Most of these patients had B-symptoms at presentation.

For histopathological examination, an excisional lymph node biopsy was performed on 38 cases (76%), and a Tru-cut lymph node biopsy was performed on 12 cases (24%). The IPS score was available only for 36 cases, among which 19 cases (53%) had low IPS (0-1), indicating a good risk group, 15 cases (42%) were in the fair risk group with IPS scores of 2-3, and 2 cases (5%) were in the poor risk group with IPS scores 4-7. Follow-up status was available only in 29 cases. The follow-up duration ranged from 20 to 75 months in these cases. Most patients (83%) survived and remained apparently healthy after treatment, 14% died, and 3% relapsed/deteriorated during the follow-up period.

### Routine diagnostic immunohistochemistry work up

Table 2 displays the percentage expression and staining intensity in HRS cells and immune cells of various subtypes of Hodgkin lymphoma. CTLA-4 IHC expression in HRS cells was negative in all cases, while in immune cells, CTLA-4 expression was observed in 45 cases (90%), with a cytoplasmic staining pattern. The expression and intensity of CTLA-4 in the immune cells of the mixed cellularity subtype showed 10-50% expression and 2+ intensity in most cases. Similarly, the expression and intensity of CTLA-4 in the immune cells of the nodular sclerosis subtype and lymphocytes-rich subtype showed 1-10% expression and 1+ intensity and 10-50% expression and 2+ intensity, respectively, in most cases. CD80 expression was present in all cases, both in HRS and immune cells. The distribution pattern in terms of percentage expression in HRS cells and immune cells in the microenvironment varied. The staining pattern was cytoplasmic in both HRS and immune cells with different intensities in various subtypes of HL. Most cases of the mixed cellularity subtype showed strong IHC expression (>50% expression with 2+ intensity) in HRS cells and moderate expression (11-50% expression with 2+ intensity) in immune cells. In lymphocyte rich subtype, most cases showed strong IHC expression in both HRS cells and immune cells. Within the microenvironment immune cells of the tumor, B7-1 expression was found in mononuclear cells, including lymphocytes, macrophages, monocytes, and dendritic cells (Tables 3 and 4).

**Table 2:** Immunohistochemical expression of CTLA-4.

			Subtype of Hodgkin lymphoma			
Cell type			Mixed cellularity	Nodular sclerosis	Lymphocyte rich	NLPHL
HRS cell			0/26	0/9	0/14	0/1
**Immune cells**	% Expression in immune cells	<1%	04	02	03	00
		1–10%	05	04	02	01
		11–50%	10	01	06	00
		>50%	07	02	03	00
	Mean intensity of IHC expression in immune cells	0	04	01	01	00
		+1	05	06	05	01
		+2	13	02	08	00
		+3	04	01	01	00

**Table 3:** Immunohistochemical expression of CD80 in HRS cells of various HL subtypes.

Expression of CD80 IHC in HRS cells of various subtypes of HL					
HRS cell	%	MC	NS	LR	NLPHL
**% Expression**	<1%	00	00	00	00
	1-10%	01	00	01	00
	11-50%	05	05	02	00
	>50%	20	04	11	1
**Mean intensity of IHC expression**	0	00	00	00	00
	+1	02	01	03	00
	+2	16	06	08	01
	+3	08	02	03	00

**Table 4:** Immunohistochemical expression of CD 80 in immune cells of various subtypes of HL.

Expression of CD80 IHC in immune cells					
Immune cells	%	MC	NS	LR	NLPHL
**% Expression in immune cells**	<1%	00	00	00	00
	1-10%	09	06	03	00
	11-50%	11	03	08	01
	>50%	06	00	03	00
**Mean intensity of IHC expression in immune cells**	0	00	00	01	00
	+1	03	04	03	01
	+2	19	05	08	11
	+3	04	00	03	02

Table 4 shows the percentage expression and staining intensity of CD80 IHC in HRS cells of various subtypes of Hodgkin lymphoma. CD80 IHC expression was cytoplasmic in HRS cells and seen in >10% of cells with a mean intensity of 2+ in most cases of HL, irrespective of the subtype. In the nodular sclerosis subtype, the distribution of CD80 expression and intensity in both HRS cells and immune cells showed that 5 out of 9 cases had 10-50% expression, and 6 out of 9 cases had 2+ intensity in HRS cells. In the immune cells, most cases showed 11-50% expression and 2+ intensity. Similarly, for the lymphocyte-rich subtype, most cases showed >50% expression and 2+ staining intensity in HRS cells and 10-50% expression and 2+ intensity in immune cells.

Table 5 shows a significant association between CTLA-4 expression in immune cells and the Ann Arbor stage at presentation. Using a cutoff expression of 10% cells, it was observed that patients with higher Ann Arbor stage (II/III/IV) had CTLA-4 expression in more than 10% of the immune cells within the tumor microenvironment, which was statistically significant.

**Table 5:** The association between CTLA-4 expression in immune cells and Ann Arbor stage of disease at presentation.

% Expression of CTLA-4 in immune cells	Ann Arbor stage I	Ann Arbor stage II	Ann Arbor stage III	Ann Arbor stage IV
**<10%**	5	5	3	1
**>10%**	1	10	7	7

Applied χ^2^ test for significance; χ^2^ value=7.964; p value=0.046 (significant).

Table 6 demonstrates the association between CTLA-4 positive immune cells and IPS score. The analysis revealed no significant association between CTLA-4 positivity in immune cells and IPS score when using a cutoff percentage of <10% and >10%.

**Table 6:** Association between CTLA-4 IHC expression in immune cells and IPS score.

% Expression of CTLA-4 in immune cells	IPS (0-1)	IPS (2-3)	IPS (4-7)
**<10%**	9	8	1
**>10%**	8	5	1

Applied χ^2^ test for significance; χ^2^ value=1.2908; p value=0.524.

Table 7 demonstrates an association between the percentage of Hodgkin Reed-Sternberg (HRS) cells and the Ann Arbor stage of disease at presentation. Patients with higher Ann Arbor stages (II/III/IV) at presentation had a greater proportion of HRS cells within the tumor microenvironment.

**Table 7:** The association between HRS cell percentage and Ann Arbor stage of disease at presentation

Ann Arbor staging	HRS cell percentage			Total
	<5%	6-10%	>10%	
**I**	4	1	1	6
	28.6%	6.2%	9.1%	14.6%
**II**	8	5	3	16
	64.3%	31.2%	27.3%	39.0%
**III**	0	7	4	11
	.0%	43.8%	36.4%	26.8%
**IV**	1	2	5	8
	7.1%	18.8%	45.5%	19.5%
**Total**	13	15	13	41
	100.0%	100.0%	100.0%	100.0%

Applied χ^2^ test for significance; χ^2^ value=18.814; p value=0.004 (significant).

Table 8 shows a significant association between the percentage of HRS cells and IPS score at presentation. As the percentage of HRS cells increases, the patient tends to present with a higher IPS score (2-3/4-7( (p value=0.001). We compared the expression of CTLA-4 and CD80 in the immune cells of the Hodgkin lymphoma microenvironment. Immune cells were categorized as T cells and antigen-presenting cells (APCs), which included macrophages, monocytes, and dendritic cells. We found that CTLA-4 expression was higher in T cells than in antigen-presenting cells (APC), and CD80 expression was higher in APC than T cells in the immune cells of the Hodgkin lymphoma microenvironment.

**Table 8:** The association between HRS cell percentage and IPS score.

IPS score	HRS cell percentage			Total
	<5%	6–10%	>10%	
**0–1**	12 (92.3%)	4 (28.6%)	3 (33.3%)	19
**2–3**	1 (7.7%)	10 (71.4%)	4 (44.4%)	15
**4–7**	0 (.0%)	0 (.0%)	2 (22.2%)	2
**Total**	13 (100.0%)	14 (100.0%)	9 (100.0%)	36

Applied χ^2^ test for significance; χ^2^ value=18.642; p value=0.001(significant).

Table 9 shows the association between CTLA-4 expression in immune cells and the Ann Arbor stage of disease at presentation. A higher percentage of CTLA-4 positive immune cells was observed in patients with advanced stage III disease in the tumor microenvironment. A higher percentage of CTLA-4 positive immune cells was observed in patients with advanced stage III disease in the tumor microenvironment.

**Table 9:** The association between CTLA-4 IHC expression in immune cells and Ann Arbor staging

CTLA4 in immune cells	Ann Arbor staging				Total
	I	II	III	IV	
**<1%**	0	0	1	0	1
	0%	0%	9.1%	0%	2.5%
**1–10%**	3	3	2	2	10
	50%	18.70%	18.2%	25.0%	25.0%
**11–50%**	2	8	3	3	16
	33.3%	50.0%	27.3%	37.5%	39.0%
**>50%**	1	5	5	3	14
	16.7%	31.3%	45.6%	37.5%	34.2%

Applied χ^2^ test for significance; χ^2^ value=2.363; p value=0.984.

Table 10 shows the relationship between CTLA-4 expressing immune cells and IPS score at presentation but does not reveal a significant association. However, when examining the expression of CTLA-4 in T cells of the tumor microenvironment, we observed a higher percentage of CTLA-4 positive T cells in patients with a high Ann Arbor stage at presentation. This increased expression of CTLA-4 in immune cells was mainly due to the higher percentage of CTLA-4 T cells in the tumor microenvironment of Hodgkin lymphoma. Similarly, we found a higher percentage of CTLA-4 positive T cells in patients with a high IPS score at presentation. However, the association between the expression of CTLA-4 in antigen-presenting cells (APC) of the tumor microenvironment and Ann Arbor stage at presentation was not significant (χ^2^ test for significance; χ^2^value=4.613; p value=0.594).

**Table 10:** The association between CD80 positive in immune cells and Ann Arbor staging.

CD80 in immune cell	Ann Arbor staging I	Ann Arbor staging II	Ann Arbor staging III	Ann Arbor staging IV	Total
**<1%**	0	0	0	0	0
**1–10%**	4	4	6	3	17
**11–50%**	1	9	2	3	15
**>50%**	1	3	3	2	9
**Total**	6	16	11	8	41

Applied χ^2^ test for significance; χ^2^ value=6.37; p value=0.702

This association demonstrates that CTLA-4-mediated suppression of antitumor T cell activation leads to a survival advantage and proliferation of immune cells in the tumor microenvironment of Hodgkin lymphoma.

**Figure 2:**
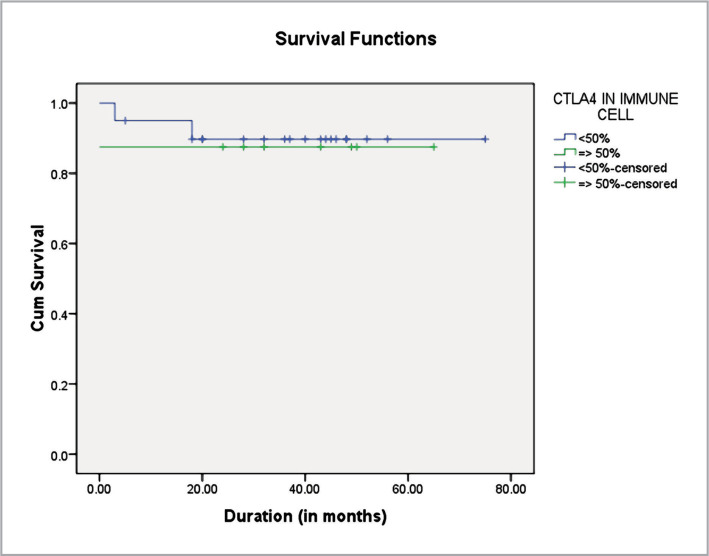
Kaplan-Meier survival curves for patients with CTLA4 expression.

The mean survival duration was longer in <50% immune cells compared to >50% groups. Overall, the mean survival was 67.633 months (Table 11, Figure 2).

**Table 11:** Kaplan-Mayer survival analysis in CTLA-4 positive immune cell

CTLA4 Expression	Estimate	Std. Error	95% CI Lower Bound	95% CI Upper Bound
**<50%**	68.392	4.450	59.670	77.113
**≥50%**	56.875	7.600	41.979	71.771
**Overall**	67.633	4.040	59.714	75.552

Estimates are limited to the largest survival time if censored. Overall comparisons were conducted using the log-rank (Mantel-Cox) test (χ^2^= 0.051, df=1, p=0.821).

**Figure 3:**
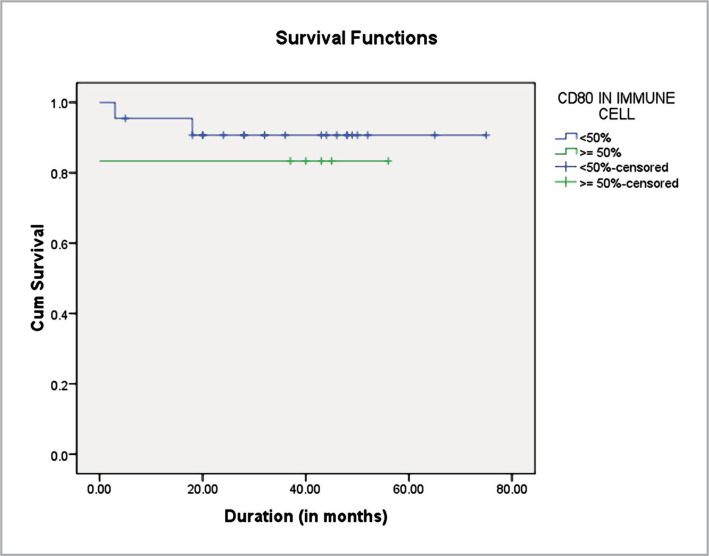
Kaplan-Meier survival curves for patients with CD80 expression.

The mean duration of survival was longer in <50% immune cells as compared to >50% groups. Overall the mean survival was 67.633±4.04 months (Table 12, Figure 3).

**Table 12:** Kaplan-Mayer survival analysis in CD80 positive immune cell

CD80 Expression	Estimate	Std. Error	95% CI Lower Bound	95% CI Upper Bound
**<50%**	69.007	4.057	61.055	76.959
**≥50%**	46.667	8.520	29.967	63.366
**Overall**	67.633	4.040	59.714	75.552

Estimates are limited to the largest survival time if censored. Overall comparisons were conducted using the log-rank (Mantel-Cox) test (χ^2^= 0.335, df=1, p=0.563)

## DISCUSSION

Hodgkin lymphoma (HL) accounts for approximately 30% of all lymphoma cases, and it is characterized by heterogeneous cellularity, with a minority of neoplastic cells and a majority of reactive non-neoplastic (immune) cells [11]. The neoplastic HRS cells are germinal centre-derived B-lymphoid cells. The non-neoplastic cells surrounding the neoplastic (HRS) cells include lymphocytes, plasma cells, neutrophils, eosinophils, and histiocytes [12]. Hodgkin lymphoma thus presents a good model to study the interactions between the tumor microenvironment and the immune interactions.

Our study included 50 cases of Hodgkin Lymphoma, of which 42 (84%) were males and 8 (16%) were females, resulting in an M: F ratio of 5.2:1. The age range of the patients was between 5 to 71 years. This ratio is higher than the M: F ratio of 2.4:1 reported worldwide in the literature. However, an overall male preponderance with an incidence of 1.2% and 0.5% in females has been reported [13]. The difference in our study may be due to the small sample size and selection bias of patients attending our tertiary care center.

Hodgkin lymphoma can occur at any age, but it is most commonly diagnosed in two age groups: individuals aged 15 to 40 (particularly those in their 20s) and those over age 55 [14]. In our study, most patients were young adults under 30 years of age, consistent with the existing literature. The mixed cellularity subtype was the most common type in our study, accounting for 26 cases (52%), followed by the lymphocyte-rich subtype, with 14 cases (28%). The nodular sclerosis subtype was observed in 9 cases (18%), and the nodular lymphocyte-predominant Hodgkin lymphoma (NLPHL) subtype was seen in only 1 case (2%).

The laboratory workup in this study involved a comprehensive review of all reported cases of Hodgkin lymphoma to confirm the diagnosis based on histomorphology and immunohistochemistry (IHC). The IHC panel included LCA, CD3, CD20, Pax5, CD15, and CD30 in most cases, and additional IHC markers such as Pan-CK, EMA, ALK, CD4, CD8, CD10, CD23, and CD68 were used to rule out other differential diagnoses.

Previous studies have shown that CTLA-4 is expressed on activated T cells among reactive immune cells in the microenvironment, and malignant HRS cells are consistently negative for CTLA-4 [58]. Similarly, in our study, CTLA-4 positivity was observed on T cells in the microenvironment, while malignant HRS cells were negative. We noted the percentage expression of CTLA-4 IHC in immune cells as <1%, 1-10%, 11-50%, and >50% and staining intensity as zero, +1, +2, and +3 along with the pattern of staining, i.e., cytoplasmic or membranous. In the mixed cellularity subtype, 10 cases (39%) showed 11-50% expression with +2 staining intensity.

In the nodular sclerosis subtype, 4 cases (45%) showed 1-10% positivity with +1 intensity, and in lymphocyte rich subtype, most cases (43%) showed 11-50% positivity with 2+ staining intensity. In our study, only 1 case of NLPHL was included, which showed weak positivity for CTLA-4. Based on the CTLA-4 IHC expression and staining results, we inferred that most cases in our study showed moderate IHC expression (11-50% expression and 2+ staining intensity) in immune cells irrespective of the subtype of classical HL. As expected, the staining pattern was cytoplasmic in all cases, and no nuclear or membranous staining patterns were seen.

B7-1(CD80) is a membrane protein present in HRS and antigen-presenting cells and is also expressed by activated B cells, monocytes, macrophages, and dendritic cells. Recent studies have shown that CD80 is also expressed by activated T cells. The interaction of CD80 with CTLA-4 on T cell subsets can enhance the immune response against tumor cells by inactivating the tumor microenvironment [15]. In this study, the IHC expression of CTLA-4 and CD80 in HRS cells and immune cells was similar to what was previously described in the literature. In a study of 12 cases of Hodgkin lymphoma, Xerri et al. observed that the majority of the population of small reactive T-lymphocytes and RS cells and variants were consistently negative for CTLA-4, but all cases showed strong immunostaining for CD80 [16]. Our study found similar results, with CTLA-4 expression observed only on immune cells, most of which were T lymphocytes, while HRS cells were consistently negative for CTLA-4.

Patients with lymphocyte predominance or nodular sclerosis frequently present with stage I-II disease and are frequently free of systemic manifestations. Most patients with disseminated disease (stages III-IV) fall under the mixed-cellularity subtype and experience constitutional symptoms like fever, night sweats, and weight loss. The International Prognostic Score (IPS), which includes the following risk factors (for each one present, the patient receives 1 point), allows for further risk stratification of patients: hemoglobin level: 10.5 g/dL; albumin level: 4 g/dL, male, under 45 years old, stage IV disease, white blood cell (WBC) count >15,000/L, which is a sign of leukocytosis. Lymphopenia is defined as a lymphocyte count of 8% of WBC count and/or an absolute lymphocyte count of 600 cells/L. Patients with advanced disease can be divided into three groups based on their IPS scores: good risk (IPS 0-1), fair risk (IPS 2-3), and poor risk (IPS 4-7).

We correlated CTLA-4 positive immune cells with IPS score and Ann Arbor stages. Cases with low IPS (good risk group) and lower Ann Arbor stage had fewer CTLA-4 positive immune cells, while those with a high IPS score and a higher stage had a higher number of immune cells, reflecting a direct correlation of average percentage of immune cells with disease outcome. Furthermore, when we set the cut-off percentage of CTLA-4 positive immune cells at 10%, a significant correlation was found (p=0.002). This correlation has not been reported in earlier literature.

We also investigated the association between the percentage of HRS cells with Ann Arbor staging and IPS score. We categorized cases based on the HRS cell percentage and found a significant association with Ann Arbor staging and IPS score. Cases with higher HRS cell percentages were significantly more likely to be in stage III/IV, whereas cases with lower HRS cell percentages tended to have lower stage (I/II) disease. This association was significant after cross-tabulation (p-value =.001). Similar to the association with Ann Arbor staging, a significant association was found between HRS cell percentage and IPS score. Cases with higher HRS cell percentages had significantly higher IPS scores as compared to the cases with lower HRS cell percentages having lower IPS scores. This showed a significant association after cross-tabulation (p-value =.004).

We observed an association between the expression of CTLA-4 in the T cell and antigen-presenting cell of the tumor microenvironment with Ann Arbor stage and IPS score at presentation. We found a significant trend showing an increase in the expression of CTLA-4 on both T cells and antigen-presenting cells as the IPS score increased. Furthermore, we followed up with patients for their disease outcome in terms of progression-free survival, relapse or recurrence, or death due to disease-related events. We obtained follow-up information for 29 patients, with a duration of follow-up ranging from 20-75 months. The 5-year survival rate for Hodgkin lymphoma is 86%, while the 10-year survival rate is 80%. These survival rates vary depending on the stage and subtype of the disease. An epidemiological study of more than 8,000 people diagnosed with Hodgkin disease between 1988 and 2001 reports a 90% 5-year survival rate for stage I, 90% for stage II, 80% for stage III, and 65% for stage IV [17]. In our study, 24 patients (83%) had progression-free survival, 1 patient (3%) relapsed or deteriorated, and 4 patients (14%) died during the follow-up period. Patients who showed disease progression /relapse or died had a high Ann Arbor stage (III/IV) and fair (IPS 2-3) or poor risk (IPS 4-7) at presentation. We analyzed the survival of patients based on the expression of CTLA-4 and CD80 in immune cells, using a cut-off value of 50% for both markers. Patients with <50% expression of CTLA-4 and CD80 had a longer mean survival duration compared to those with >50% expression. The overall mean progression-free survival was 67.633±4.04 months.

## CONCLUSION

This study provides pioneering insights into the expression of immune inhibitory molecules CTLA-4 and B7-1 in Hodgkin lymphoma, with no published data from India to compare these findings in our population. In this study, we analyzed the expression and potential role of CTLA-4 and CD80 signaling pathways related to immune suppression and disease progression in the tumor microenvironment of various subtypes of Hodgkin Lymphoma. Further studies are needed to substantiate our findings.

Considering our results on CTLA-4 expression in the immune cell microenvironment and the availability of targeted drugs such as Ipilimumab, which acts through CTLA-4 blockade, it may be relevant to explore this as a targeted therapy for HL cases, particularly for those with refractory disease unable to achieve disease cure before ASCT, for patients relapsing after ASCT, and for whom no further curative options are currently available.

## References

[1] Lamprecht B, Kreher S, Anagnostopoulos I, Jöhrens K (2008). Aberrant expression of the Th2 cytokine IL-21 in Hodgkin lymphoma cells regulates STAT3 signaling and attracts Treg cells via regulation of MIP-3alpha. Blood.

[2] Diehl V, Thomas RK, Re D (2004). Part II: Hodgkin's lymphoma--diagnosis and treatment. Lancet Oncol.

[3] Aldinucci D, Lorenzon D, Olivo K, Rapanà B, Gattei V (2004). Interactions between tissue fibroblasts in lymph nodes and Hodgkin/Reed-Sternberg cells. Leuk Lymphoma.

[4] Aldinucci D, Olivo K, Lorenzon D, Poletto D (2005). The role of interleukin-3 in classical Hodgkin's disease. Leuk Lymphoma.

[5] Aldinucci D, Lorenzon D, Cattaruzza L, Pinto A (2008). Expression of CCR5 receptors on Reed-Sternberg cells and Hodgkin lymphoma cell lines: involvement of CCL5/Rantes in tumor cell growth and microenvironmental interactions. Int J Cancer.

[6] Cattaruzza L, Gloghini A, Olivo K, Di Francia R (2009). Functional coexpression of Interleukin (IL)-7 and its receptor (IL-7R) on Hodgkin and Reed-Sternberg cells: Involvement of IL-7 in tumor cell growth and microenvironmental interactions of Hodgkin's lymphoma. Int J Cancer.

[7] Rudd CE, Taylor A, Schneider H (2009). CD28 and CTLA-4 coreceptor expression and signal transduction. Immunol Rev.

[8] Yokosuka T, Kobayashi W, Takamatsu M, Sakata-Sogawa K (2010). Spatiotemporal basis of CTLA-4 costimulatory molecule-mediated negative regulation of T cell activation. Immunity.

[9] Ribas A (2012). Tumor immunotherapy directed at PD-1. N Engl J Med.

[10] Murakami N, Riella LV (2014). Co-inhibitory pathways and their importance in immune regulation. Transplantation.

[11] Jaffe ES, Harris NL, Stein H, Vardiman JW (2001). Tumours of haematopoietic and lymphoid tissues.

[12] Diehl V (2007). Hodgkin's disease--from pathology specimen to cure. N Engl J Med.

[13] Doll R, Muir C, Waterhouse J (1970). Cancer incidence in five continents.

[14] Kumar V, Abbas AK, Aster JC (2020). Robbins &Cotran Pathologic Basis of Disease.

[15] Delabie J, Ceuppens JL, Vandenberghe P, de Boer M (1993). The B7/BB1 antigen is expressed by Reed-Sternberg cells of Hodgkin's disease and contributes to the stimulating capacity of Hodgkin's disease-derived cell lines. Blood.

[16] Xerri L, Devilard E, Hassoun J, Olive D, Birg F (1997). In vivo expression of the CTLA4 inhibitory receptor in malignant and reactive cells from human lymphomas. J Pathol.

[17] National Cancer Institute Surveillance. Epidemiology and End Results database.

